# A Rare Malignant Disease, Dermatofibrosarcoma Protuberans of the Breast: A Retrospective Analysis and Review of Literature

**DOI:** 10.1155/2020/8852182

**Published:** 2020-11-09

**Authors:** Yihua Wang, Yu Wang, Rui Chen, Zhenrong Tang, Shengchun Liu

**Affiliations:** ^1^Department of Endocrine and Breast Surgery, The First Affiliated Hospital of Chongqing Medical University, Chongqing 400016, China; ^2^Department of Thyroid and Breast Surgery, The Affiliated Hospital of Zunyi Medical University, Zunyi, 563000 Guizhou, China

## Abstract

Dermatofibrosarcoma protuberans (DFSP) is a rare low-grade fibroblastic mesenchymal tumor derived from the dermis. The aim of this retrospective analysis was to summarize the clinicopathological data from our cases and published cases to offer more evidence for the recognition of dermatofibrosarcoma protuberans (DFSP). A total of 6 breast DFSP patients who had received treatment in our hospital were retrospectively enrolled, and detailed clinicopathological data were gathered for analysis. The median age was 29.5 years (ranging from 17 to 42 years). Most cases presented a red or brown-red, mobile, well-circumscribed, protruding, breast mass (ranging from 1 to 3 cm). For histopathology, all cases (6/6) showed a storiform pattern of spindle cells that were positive for CD34 (6/6) and Vimentin (5/6) and negative for smooth muscle actin (0/6) and S-100 protein (0/6). The majority of patients (5/6) underwent wide local excision, with 2 cases treated with radiotherapy. With a median follow-up of 36 months, all 6 patients survived without recurrence or metastasis. The PubMed database was used to search for similar cases. Eventually, 36 cases were included in this review, while cases without detailed clinical information or not reported in English were excluded from the analysis. To summarize, DFSP of the breast is an extremely rare malignancy characterized by spindle tumor cells arranged in a storiform pattern and positivity for CD34. The core needle biopsy is one of the crucial methods for its preoperative diagnosis. Management of DFSP is mainly based on surgical excision. It is prone to local recurrence, so long-term follow-up is required.

## 1. Introduction

Dermatofibrosarcoma protuberans (DFSP) is a rare low-grade fibroblastic mesenchymal tumor derived from the dermis [1]. It was first described by Darier and Ferrand in 1924 as a progressive and recurrent dermatofibroma [2] and termed by Hoffmann [3] in 1925. The lesion corresponds to approximately 1% of all soft tissue sarcomas and less than 0.1% of all malignancies, with an annual incidence of 4.2-4.5 cases per million [4, 5]. It occurs most frequently between the second to fifth decades of life and typically appears in the dermis and subcutis [6, 7]. DFSP can be all over the body; the most common site is the trunk (42-72%), followed by the proximal extremities (16-30%) [6, 8], and breast involvement is uncommon [8, 9]. Due to the rarity of breast DFSP cases, the current understanding of DFSP of the breast is still inadequate. Hence, we present our own data on 6 patients with breast DFSP, including clinicopathological features, therapeutic strategies, and prognostic significance, and summarize the clinicopathological data from published cases to offer more evidence for the recognition of this tumor.

## 2. Materials and Methods

**Figure 1:**
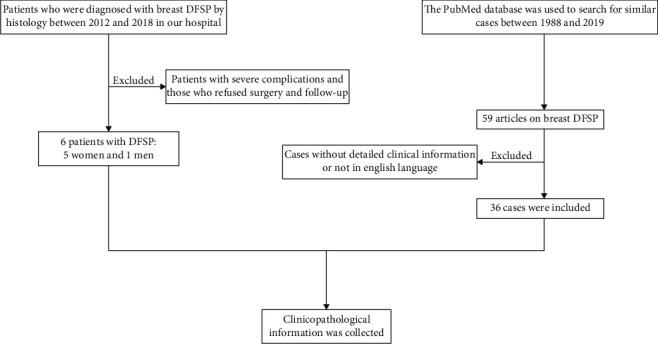
The flow chart for the synopsis of the Material and Methods section.

The flow chart for the Material and Methods section was provided in Figure 1.

### 2.1. Patients

This was a retrospective analysis, in which we included breast tumor patients who were diagnosed with DFSP by histology and had received treatment in The First Affiliated Hospital of Chongqing Medical University between 2012 and 2018. Clinicopathological information was collected by consulting the medical records. Patients with severe complications who could not be treated surgically and those who refused surgery and follow-up treatment were excluded from the analysis. This research was conducted ethically in accordance with the World Medical Association Declaration of Helsinki and was approved by the Ethics Committee of The First Affiliated Hospital of Chongqing Medical University, who deemed that written informed consent was not necessary due to the retrospective nature of the research.

### 2.2. Patient and Public Involvement

No patients or members of the public were involved in this study.

### 2.3. Diagnostics and Therapies

Ultrasonography and laboratory tests were routinely performed for further diagnosis. Most patients underwent a preoperative excision biopsy (EB). The gold standard for the diagnosis of breast DFSP depends on histopathology and immunohistochemistry (IHC). All patients received surgical treatment.

### 2.4. Histopathology

The histological slides were reviewed and classified according to the 2012 WHO classification [10]. We investigated the cell morphology and distribution of breast DFSP by hematoxylin-eosin staining and the expression of important markers correlated with breast tumors by immunohistochemical staining, including a series of makers: (1) cluster of differentiation 34 (CD34), as a specific biomarker of vascular endothelial cells, is closely associated with the status of neovascularization during the process of tumor growth and thus is sensitive to tumor angiogenesis [11]. (2) Cluster of differentiation 68 (CD68), one of the members from the growing family of hematopoietic mucin-like molecules known as lysosome/endosome-associated membrane glycoproteins (LAMPs), is highly expressed in human monocytes and tissue macrophages. CD68 has been universally used as a pan-macrophage or M1 macrophage marker as it has been reported as one of the most common markers of tumor-associated macrophages (TAM) [12]. (3) Vimentin, also known as fibroblast intermediate filament, anchors and supports organelles within the cytosol of mesenchymal cells. This protein is upregulated during epithelial to mesenchymal transition (EMT), a process that often occurs in cancer metastasis, and contributes to EMT by changing cell shape and motility. Previous studies have shown that Vimentin is a metastasis-associated factor in multiple malignancies, such as breast and prostate cancer. Current thought on Vimentin is that it may serve as a potential biomarker for metastasis and play an important part in tumor progression [13]. (4) Smooth muscle actin (SMA), as an isoform of actin, predominates among vascular smooth muscle cells (SMC) with an important role in mechanotransduction and generation of traction forces in SMC, and it is of great importance for fibrogenesis as it has been employed as a marker for a subset of activated fibrogenic cells, myofibroblasts [14]. (5) Epithelial membrane antigen (EMA), attributed to a heterogeneous group of heavily glycosylated proteins, has been shown to express in most normal and epithelial neoplastic cells. EMA has been used as one of the markers of epithelial cells, particularly the luminal cells, of salivary gland tumors [15]. (6) Cytokeratin (CK) is a global term for the family of intermediate filament proteins of epithelial origin; the modality of CK expression may help differentiate colorectal from lung carcinomas based on low and high molecular weight types [16]. Cytokeratins have been extensively used as one of the markers for disease progression in cancer patients. (7) S-100 protein (S100), a dimer intracellular calcium-binding protein, has been implicated in neuronal proliferation and differentiation [17]. The protein has been reported to be associated with several tumors, such as melanoma and highly differentiated neuroblastomas [18]. (8) Ki67 is a nonhistone nuclear protein present during all active phases of the cell cycle, but absent in resting G0-stage cells [19]. Expression of the Ki67 protein plays an important role in the proliferative activity of intrinsic cell populations in malignant tumors, allowing it to be used as a marker of tumor aggressiveness as shown in malignancies of the breast, soft tissue, lung, prostate, cervix, and central nervous system [20].

### 2.5. Follow-Up

Follow-up investigations, including a clinical examination and a radiological assessment, were performed in regular intervals (3-month intervals in years 1-3, 6-month intervals in years 4-5, and 12-month intervals in years 6-10 after diagnosis). The detailed information of patients with recurrence and metastasis and the survival rate were recorded truthfully. The deadline for follow-up was December 31, 2018.

## 3. Results

### 3.1. Basic Information

Between 2012 and 2018, a total of 6 breast tumor patients with DFSP were enrolled in our study for further analysis (5 were female, and 1 was male). The median age was 29.5 years (ranging from 17 to 42 years, mean age 29.7 years). All patients visited the hospital due to a palpable breast mass (ranging from 1 to 3 cm, mean 2.25 cm).

### 3.2. Physical Examination

Most patients (5/6) had primary tumors characterized by a red or brownish red (*n* = 4), protruding (*n* = 4), well-circumscribed (*n* = 4), and firm (*n* = 5) nodule. Only one patient suffered from a recurrent tumor that could be palpable in the surgical scar. All the lumps were painless and mobile. There was no clinical evidence of axillary or supraclavicular lymph node swelling (Table 1).

### 3.3. Imaging Examination

**Figure 2:**
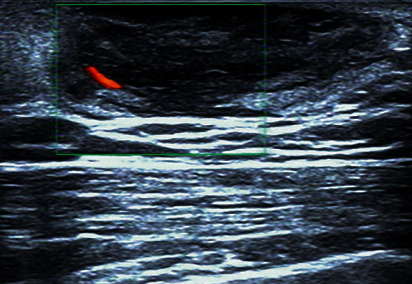
The typical ultrasound image of dermatofibrosarcoma protuberans of the breast. Note: ultrasound shows a well-defined hypoechoic mass in the subcutaneous tissue with a slightly hyperechoic surrounding area. Increased internal vascularity of the lesion is demonstrated by color Doppler scanning.

On ultrasound examination, the tumors were visualized as low-echoic (*n* = 5) or mixed-echoic (*n* = 1) lesions (Figure 2), which were located in the subcutaneous tissue and partly surrounded by a slightly high-echoic area (*n* = 2). Upon color Doppler scanning, short cord-like blood flow signals were detected in 2 patients. One case underwent mammography which suggested a circumscribed, round, radiopaque lesion with a sharp contour. Due to a superficial location characteristic of the lesion, however, none received computed tomography or magnetic resonance imaging (MRI) examination.

### 3.4. Provisional Diagnosis

The majority of patients (5/6) were empirically diagnosed with benign lesions at the outpatient visit. At the initial stage, 3 were diagnosed with fibroadenoma, 1 was diagnosed with dermatofibroma, and 1 was diagnosed with hemangioma.

### 3.5. Pathology and Immunohistochemistry

**Figure 3:**
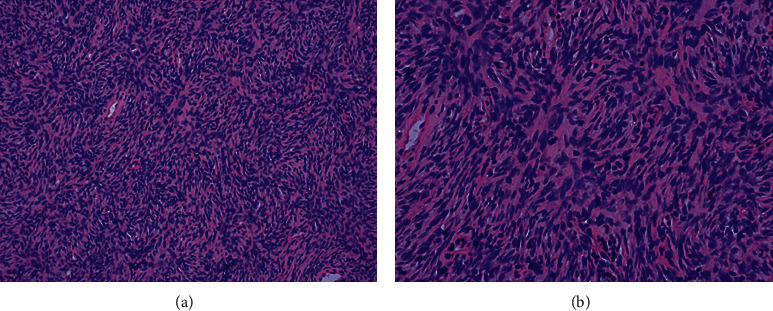
Hematoxylin-eosin staining of dermatofibrosarcoma protuberans of the breast. Note: the characteristic storiform pattern of spindle cells is shown with hematoxylin and eosin staining. (a) 200x. (b) 400x.

**Figure 4:**
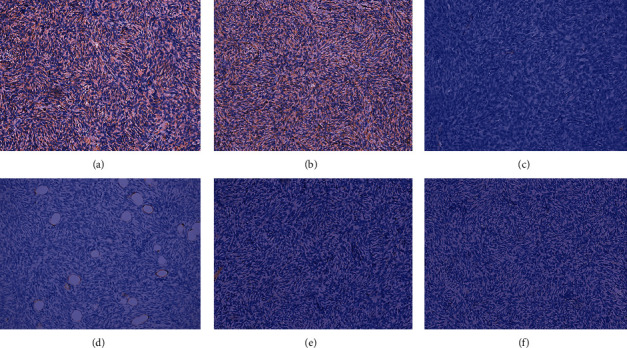
Immunohistochemistry of the breast dermatofibrosarcoma protuberans. Note: tumor immunohistochemistry shows CD34 positivity ((a) 200x) and Vimentin positivity ((b) 200x). Besides, smooth muscle actin ((c) 200x), S-100 protein ((d) 200x), cytokeratin ((e) 200x), and epithelial membrane antigen ((f) 200x) are negative in tumor cells.

Pathological examinations were performed in all 6 cases. Gross specimens yielded homogeneous, off-white (*n* = 4) or taupe (*n* = 1) masses with focal necrosis (*n* = 3). Histologically, the storiform pattern of spindle cells (*n* = 6) (Figures 3(a) and 3(b)) infiltrating into subcutaneous tissue (*n* = 5) above the mammary gland was noticed. Regarding IHC, the expression rates of CD34 (Figure 4(a)), Vimentin (Figure 4(b)), SMA (Figure 4(c)), S-100 (Figure 4(d)), CK (Figure 4(e)), and EMA (Figure 4(f)) were 6/6, 5/6, 0/6, 0/6, 0/4, and 0/3, respectively. The Ki67 index showed frequent positivity, fluctuating from 1% to 20% (Table 2).

### 3.6. Treatment

Most patients (5/6) underwent an EB and were diagnosed with DFSP following a pathological examination. Then, they underwent wide local excision (WLE) (*n* = 5) or mastectomy (*n* = 1) because of residual disease (*n* = 2) or undetected margins (*n* = 3). Moreover, an intraoperative frozen section examination was performed to confirm no residual tumor at the incisal margin. Another one underwent WLE without a preoperative biopsy due to the clinical suspicion of recurrent DFSP (Table 3). Some patients (2/6) were recommended for radiotherapy in view of the nature of rapidly growing (*n* = 1) or locally recurrent (*n* = 1) DFSP. None received chemotherapy (Table 3).

### 3.7. Prognosis

The median follow-up period was 36 months (ranging from 18 to 56 months). All 6 patients survived without recurrence or metastasis during the follow-up period (Table 3).

### 3.8. Literature Review

The PubMed database was used to search for similar cases. Between 1988 and 2019, there were 59 articles reporting on breast DFSP. Cases without detailed clinical information or not reported in English were excluded from the analysis. Eventually, 36 cases were included in this review.

### 3.9. Clinical Presentation

The clinical features of patients with breast DFSP from reported cases were shown in Table 4. The median age of the patients presenting with DFSP of the breast was 39 years (ranging from 2 to 102 years), and the female to male ratio was 31 : 5. Generally, patients presented with a slowly enlarging, firm, mobile, well-circumscribed mass (ranging from 1 to 12 cm in size) as shown in Table 4. Lesions were often accompanied by skin changes, such as red or brown coloring (*n* = 23), protrusion (*n* = 19), ulceration (*n* = 6), erythematous (*n* = 5), and skin retraction (*n* = 2). On the contrary, 6 patients had no skin changes. None was reported with associated lymphadenopathy.

### 3.10. Pathological Presentation

Histologically, the majority of breast DFSP patients presented (Table 5) with spindle cells (34/36) arranged in a storiform pattern (30/36). Apart from the unavailable 6 cases, most tumors involved the dermis (26/30) and subcutis (29/30) with some cases involving adipose tissue (16/30), mammary gland (3/30), and muscle (1/30). As listed in Table 5, CD34 was the most commonly positive immunohistochemical marker (32/32, 4 were not available), with an 8/8 expression rate for Vimentin (28 were not available). The negative rates were 8/9, 17/17, 10/10, 14/17, and 5/5 for Desmin (28 were not available), S100 (19 were not available), CK (26 were not available), SMA (19 were not available), and EMA (31 were not available), respectively. Additionally, among the 5 cases of breast DFSP with genetic information, 4 cases presented the collagen type I *α* 1 (COL1A1)-platelet-derived growth factor (PDGF) *β* fusion gene, which often accompanies a chromosomal translocation involving 17q22 (COL1A1 at 17q22) and 22q13 (PDGF*β* at 22q13) and a ring chromosome formation; the protein product of COL1A1-PDGF*β* fusion gene binds to the PDGF receptor and further stimulates the growth of DFSP cells by autocrine secretion [21]. 1 presented the PDGF*β* gene rearrangement.

### 3.11. Diagnosis, Treatment, and Outcomes

The diagnosis and treatment information of patients with DFSP of the breast from reported cases was listed in Table 6. Thirty-three patients were diagnosed with DFSP, while other three were diagnosed with the fibrosarcomatous transformation of DFSP (DFSP-FS). The ratio of primary to recurrent tumors was 30 : 6. Most patients underwent preoperative biopsies (10 for EB, 9 for core needle biopsies (CNB), 2 for punch biopsies, and 7 for fine needle aspirations (FNA). Of 7 patients who underwent FNA, 5 underwent additional biopsies, such as EB (*n* = 3) and CNB (*n* = 2). Surgery was the main treatment (35/36). Twenty-five cases (69.4%) were treated with WLE, while 7 patients (19.4%) were treated with mastectomy. Moreover, 8 patients (22.2%) were treated with postoperative radiotherapy. Follow-up data were provided for 20 patients. In the median follow-up period of 12 months (ranging from 6 to 70 months), no recurrence or metastasis was reported.

## 4. Discussion

DFSP of the breast is considered a low-grade, slowly growing tumor and spans years or decades. It has similar clinical characteristics to lesions on other sites. In our review, the median patient age at presentation was 39 years, and the median size of the tumor was 35 mm. In the early stages, this lesion is characterized by a red or brown-red, mobile, well-defined superficial nodule surrounded sometimes by hemangiectasis [22, 23], which can be confused with benign lesions, such as dermatofibromas and keloids. As the disease progresses, the tumor gradually appears as a reddish, symptomatic, protruding multinodular mass with an irregular border [24–26]. In a few cases, DFSP of the breast presented as a single, painless, well-defined deep mass with no skin changes [27–29]. It is necessary to strengthen its differentiation with breast fibroadenomas and phyllodes tumors. Upon ultrasound exploration or mammography, the image resembles that of a benign breast tumor. MRI may help to assess the extent of tumor infiltration prior to surgery [22, 30, 31].

Clinical suspicion must be confirmed by pathology before definitive surgery. A punch or an excisional biopsy, preferably of a deep subcutaneous layer, is strongly recommended for DFSP [21]. Wide undermining is discouraged, because it is not conducive to the pathological diagnosis of reexcision margins and may lead to tumor seeding [32]. For DFSP of the breast, CNB is an effective way to preliminarily diagnose DFSP. This approach is less traumatic and allows sufficient specimens to determine the cell morphology and the response to immunohistochemical staining [22, 24, 27]. FNA does not seem to apply to DFSP of the breast, because it is difficult to obtain sufficient tissues [28, 33].

DFSP diagnosis depends on histopathology and immunohistochemistry [21]. Breast DFSP often presents as a solid tumor located in the dermis and subcutis, infiltrating into adipose tissue, even glandular tissue and muscles [21, 27, 34]. The phenomenon may help to differentiate DFSP from some benign tumors located in the dermis, such as dermatofibromas and keloids. Moreover, on histopathological examination, DFSP of the breast usually shows a marked storiform pattern of spindle-shaped cells. This is significantly different from phyllodes tumors, which present with a biphasic pattern composed of spindle cells around ducts. CD34 has been considered a vital marker for DFSP and can be used to differentiate DFSP of the breast from CD34-negative fibrous soft tissue tumors, such as dermatofibromas, breast fibroadenomas, and fibrosarcomas. Nonetheless, it is worth noting that CD34 may be reduced or absent in areas of fibrosarcomatous transformation [23, 33, 35]. Fibrosarcoma is a high-grade soft sarcoma with increased cellular fibroblastic proliferation in the herringbone pattern with atypia and mitoses [36]. DFSP is often positive for Vimentin and negative for other routinely tested markers, including S100, SMA, CK, EMA, Desmin, CD68, and XIIIa. These immunohistochemical indicators may help to exclude myoepithelioma, fibromatosis-like metaplastic carcinoma, and so on. When difficult to diagnose, FISH analysis of the COL1A1-PDGF*β* fusion gene using routine biopsy specimens is a quick and convenient method [37]. In reported 5 cases of breast DFSP with gene detection, 4 cases presented the COL1A1-PDGF*β* fusion gene [9, 23, 38, 39], another presented the PDGF*β* gene rearrangement [40].

The treatment, for DFSP of the breast, is based on surgery, whether primary or recurrent. The principal aim of surgery is to remove the tumor completely, because of the close relationship between residual tumor and local recurrence [41–43]. WLE is a very reasonable approach that has been widely used worldwide for DFSP of the breast. It is important to define the optimal surgical margin width around the primary tumor. To achieve histological margin control, both the National Comprehensive Cancer Network guidelines and the S1 guidelines call for at least 2 cm surgical margins investing the fascia of the muscle or pericranium [32, 44]. Most cases of breast DFSP followed guidelines. A few cases indicated no local recurrence or metastasis with a surgical margin width of less than 2 cm [29, 45, 46]. Nevertheless, most of them reported a short follow-up period. In our experience, WLE involved a minimum surgical margin of 2 cm first. If any margins were positive on multiple frozen section margins, we excised an additional 1 cm along that margin and performed frozen sections to obtain disease-free margins. Mohs micrographic surgery (MMS) is a stepwise procedure of tumor excision and usually performed as an outpatient with local anesthesia; this technique is conducted with mapping and histopathologic biopsy of all surgical margins with tangential frozen sections by the Mohs surgeon; if residual tumor cells are confirmed, further wider and/or deeper reexcision of another layer of surrounding tissues is performed [47]. This process is repeated until 100% of the tumor margins are free of tumor cells. Such a new novel technique is an ideal surgical approach, especially for lesions on the face, scalp, or neck, that offers the advantage of an immediate examination of the microscopic margin and the protection of healthy tissue [48]. This approach may apply to lesions in the breast due to the ability to spare a considerable amount of tissue with less impact on the shape of the breast. Unfortunately, no case has been reported for this technique in DFSP of the breast.

Multiple studies have shown that DFSP is a radioresponsive tumor [41, 49]. For recurrent tumors or unresectable residual lesions, adjuvant radiotherapy is recommended in order to control the progression of disease [32, 49, 50]. Moreover, in a meta-analysis including 167 DFSP patients treated with adjuvant radiotherapy, Chen et al. [51] demonstrated that adjuvant radiotherapy might be considered for all patients undergoing surgical excision regardless of the surgical margin. Some cases of breast DFSP with negative margin reported the use of postoperative radiotherapy, but no long-term follow-up data included. It is reported that most DFSP have the translocation t(17; 22) (q22; q13), resulting in the COL1A1-PDGF*β* fusion gene, which provides a biological basis for treatment with tyrosine kinase inhibitors, such as imatinib [52]. In the advanced or metastatic group, imatinib therapy is warranted if a unique translocation is confirmed [53, 54]. In a systematic review including 152 locally advanced or metastatic DFSP patients who received imatinib treatment, the results found that imatinib is a significantly effective therapy in these patients, with a complete response seen in 5.2% of patients and a partial response in 55.2% [53].

DFSP has a high tendency of local recurrence and a low propensity for metastasis [21, 55, 56]. Most local recurrences occur within 3 years of surgery; although, late recurrences have also been reported [57, 58]. So long-term follow-up examinations are recommended for DFSP of the breast cases. Advanced age, a large tumor size, the DFSP-FS subtype, and the narrowed margin of resection may be the adverse prognostic features of DFSP [42, 59, 60].

## 5. Conclusion

DFSP of the breast has similar clinical characteristics to DFSP at other sites. The CNB is one of the crucial methods for its preoperative diagnosis. Surgical excision with at least 2 cm margins may reduce the risk of recurrence. Radiotherapy and imatinib therapy are beneficial for disease control. DFSP is prone to local recurrence; hence, long-term follow-up is required.

## Figures and Tables

**Table 1:** Clinical features of breast dermatofibrosarcoma protuberans patients at diagnosis in our hospital.

No.	Gender	Age (y)	Size (cm)	P/R	Mobility	Margins	Skin changes	Echogenicity	CFDI	Density
1	Male	27	3∗2	P	Mobile	Well-circumscribed	Red, protruding	Hypoechoic	P	NA
2	Female	40	3∗2	R	Mobile	Irregular	Protruding	Mixed echoic	N	NA
3	Female	42	1∗1	P	Mobile	Well-circumscribed	Brownish red	Hypoechoic	P	Hyperdense
4	Female	32	2∗1	P	Mobile	Irregular	Red, protruding	Hypoechoic	N	NA
5	Female	20	2∗1	P	Mobile	Well-circumscribed	No changed	Hypoechoic	N	NA
6	Female	17	2.5∗2	P	Mobile	Well-circumscribed	Red, protruding	Hypoechoic	P	NA

Abbreviations: P/R: primary/recurrent; CDFI: color Doppler flow imaging; P: positive; N: negative; NA: unavailable.

**Table 2:** Pathological features of breast dermatofibrosarcoma protuberans patients in our hospital.

No.	Tumor color	Tumor texture	Cell morphology	CD34	S100	SMA	Vimentin	CD68	CK	EMA	Desmin	Ki67 (%)
1	Off-white	Unknown	Spindle cells	P	N	N	P	P	N	N	N	10
2	Off-white	Hard	Spindle cells	P	N	N	P	NA	N	P	N	10
3	Off-white	Rubbery	Spindle cells	P	N	N	P	N	N	NA	NA	<5
4	Unknown	Unknown	Unknown	P	N	N	P	N	N	N	NA	20
5	Taupe	Rubbery	Spindle cells	P	N	N	N	P	NA	NA	NA	1
6	Unknown	Unknown	Spindle cells	P	N	N	P	N	NA	NA	NA	10

Abbreviations: CD: cluster of differentiation; S100: S-100 protein; SMA: smooth muscle actin; CK: cytokeratin; EMA: epithelial membrane antigen; P: positive; N: negative; NA: unavailable.

**Table 3:** Therapy and follow-up of breast dermatofibrosarcoma protuberans patients in our hospital.

No.	Procedure	MW (cm)	RT	CT	Follow-up (m)	R/M	Survival
1	Mastectomy	NA	No	No	42	No	Yes
2	WLE	3	Yes	No	40	No	Yes
3	WLE	4	No	No	38	No	Yes
4	WLE	3	No	No	56	No	Yes
5	WLE	2	No	No	22	No	Yes
6	WLE	2.5	Yes	No	18	No	Yes

Abbreviations: MW: margin width; RT: radiotherapy; CT: chemotherapy; R/M: recurrence/metastasis; WLE: wide local excision; NA: unavailable.

**Table 4:** Clinical features of patients diagnosed with dermatofibrosarcoma protuberans of the breast from reported cases.

Variable	*N* (%)
Gender	
Male	5 (16.1%)
Female	31 (83.9%)
Size (cm)	
<2	5 (16.1%)
2-5	18 (36.0%)
>5	12 (33.3%)
Unknown	1 (2.8%)
Age (y)	
<20	4 (11.1%)
20-50	25 (69.4%)
>50	6 (16.7%)
Unknown	1 (2.8%)
Tumor presentation^∗^	
Firm	15 (41.7%)
Mobile	18 (36.0%)
Well-circumscribed	11 (30.6%)
Irregular	5 (16.1%)
Skin changes^∗^	
Red or brown	23 (63.9%)
Protruding	19 (52.8%)
Erythematous	5 (16.1%)
Ulceration	6 (16.7%)
No changed	6 (16.7%)
Retraction	2 (5.6%)

Notes: ^∗^percentage of all cases reporting any clinical presentation data.

**Table 5:** Histologic characteristics of patients diagnosed with dermatofibrosarcoma protuberans of the breast from reported cases.

Variable	*N* (%)
Infiltration^∗^ (*n* = 30)	
Dermis	26 (86.7%)
Subcutis	29 (96.7%)
Adipose tissue	16 (53.3%)
Breast tissue	3 (10.0%)
Muscular layer	1 (3.0%)
Histology^†^ (*n* = 36)	
Spindle cells	34 (94.4%)
Storiform pattern	30 (83.3%)
Immunostaining^†^	
CD34 (+) (*n* = 32)	32 (100.0%)
Vimentin (+) (*n* = 8)	8 (100.0%)
Desmin (-) (*n* = 9)	8 (88.9%)
S100 (-) (*n* = 17)	17 (100.0%)
CK (-) (*n* = 10)	10 (100.0%)
SMA (-) (*n* = 17)	14 (82.4%)
EMA (-) (*n* = 5)	5 (100.0%)
XIIIa (-) (*n* = 4)	4 (100.0%)
Bcl-2 (-) (*n* = 4)	4 (100.0%)

Notes: ^∗^percent of all cases reporting any data of tumor infiltration. ^†^Percentage of all cases reporting any pathological data. Abbreviations: CD: cluster of differentiation; S100: S-100 protein; CK: cytokeratin; SMA: smooth muscle actin; EMA: epithelial membrane antigen.

**Table 6:** Diagnosis and treatment of patients diagnosed with dermatofibrosarcoma protuberans of the breast from reported cases.

Variable	*N* (%)
Diagnosis	
DFSP	33 (91.7%)
DFSP-FS	3 (8.3%)
Primary or recurrent	
Primary	30 (83.3%)
Recurrent	6 (16.7%)
Preoperative biopsy	
CNB	7 (19.4%)
Excision biopsy	10 (27.7%)
Punch biopsy	2 (5.6%)
FNA	2 (5.6%)
FNA-core biopsy	2 (5.6%)
FNA-excision biopsy	3 (8.3%)
No biopsy	10 (27.8%)
Operation	
WLE	25 (69.4%)
Mastectomy	7 (19.4%)
LE	1 (2.8%)
No operation	1 (2.8%)
Unknown	2 (5.6%)
Postoperative radiotherapy	
Yes	8 (22.2%)
No	28 (77.8%)

Abbreviations: DFSP: dermatofibrosarcoma protuberans; DFSP-FS: the fibrosarcomatous transformation of DFSP; CNB: core needle biopsy; FNA: fine needle aspiration; WLE: wide local excision; LE: local excision.

## Data Availability

The datasets used and/or analyzed during the current study are available from the corresponding author on reasonable request.

## Abbreviations

DFSP: Dermatofibrosarcoma protuberans
EB: Excision biopsy
IHC: Immunohistochemistry
CD: Cluster of differentiation
LAMPs: Lysosome/endosome-associated membrane glycoproteins
TAM: Tumor-associated macrophages
EMT: Epithelial to mesenchymal transition
SMA: Smooth muscle actin
SMC: Smooth muscle cells
EMA: Epithelial membrane antigen
CK: Cytokeratin
S100: S-100 protein
MRI: Magnetic resonance imaging
WLE: Wide local excision
COL1A1: Collagen type I *α* 1
PDGF: Platelet-derived growth factor
DFSP-FS: The fibrosarcomatous transformation of DFSP
CNB: Core needle biopsy
FNA: Fine needle aspiration
MMS: Mohs micrographic surgery.
